# Morphology of Maxillary Central Incisors in a Mixed Swiss–German Population by Means of Micro-CT

**DOI:** 10.3390/dj13020072

**Published:** 2025-02-05

**Authors:** Thomas Gerhard Wolf, Kevin Simon Florian Ottiger, David Donnermeyer, Sven Schumann, Andrea Lisa Waber

**Affiliations:** 1 Department of Restorative, Preventive and Pediatric Dentistry, School of Dental Medicine, University of Bern, 3010 Bern, Switzerlanddavid.donnermeyer@unibe.ch (D.D.);; 2 Department of Periodontology and Operative Dentistry, University Medical Center of the Johannes Gutenberg University Mainz, 55131 Mainz, Germany; 3 Institute for Anatomy, Johannes Gutenberg University Mainz, 55128 Mainz, Germany; 4 Institute of Anatomy, Brandenburg Medical School, 16816 Neuruppin, Germany

**Keywords:** accessory canals, internal morphology, maxillary central incisors, root canal configuration, micro-computed tomography

## Abstract

**Background/Objectives**: The objective of this study was to investigate the internal morphology and root canal configurations (RCCs) of maxillary central incisors (MxCIs) in a Swiss–German population by means of micro-computed tomography (µCT). **Methods**: RCCs, main foramina, and accessory canals of MxCIs were examined using µCT and 3D imaging software. The root canal anatomy was classified according to three classification systems by Vertucci (Ve, 1984), Weine et al. (We, 1969), and Briseño-Marroquín et al. (Br, 2015). **Results**: The most common RCC observed among a total of 112 investigated single-rooted maxillary central incisors was Br 1-1-1/1 (97.3%, Ve I, We I), with a small percentage showing Br 1-1-1/2 (2.7%). One main foramen existed in 87.5% of the specimens, 8% had one accessory foramen, 3.5% had two, and a rare case had four accessory foramina (0.9%). Accessory root canals were mainly located in the middle and apical regions of the roots. **Conclusions**: Detailed insights into the root canal morphology of MxCIs in a Swiss–German population are provided. The predominant RCC was a simple root canal (Ve I, Br 1-1-1/1). However, accessory canals were detected in the middle and apical third in over 40% of the teeth examined. These anatomical features should be considered during endodontic treatment planning and execution.

## 1. Introduction

Orthograde endodontic treatments are considered a reliable method of tooth preservation, with success rates ranging from 86% to 98% [1]. Nonetheless, instances of failure have been documented in certain cases [2]. The most common reason for unsuccessful endodontic treatment is the existence of bacteria within the root canal system [2,3]. The long-term success of the treatment is contingent upon a precise comprehension of the morphology of the root canal system [4]. This knowledge allows the practitioner to prepare the tooth in an optimal manner, to clean it thoroughly, both mechanically and chemically, and to seal the canal system completely in the apical area [1,2,3,4]. Each tooth presents unique challenges regarding preparation, disinfection, and obturation. The considerable diversity of root canal morphology demonstrates that, while a standardized approach provides the foundation for success, it is not a sufficient means of adequately treating complex anatomical conditions. It is imperative to possess a comprehensive grasp of the potential root canal configurations (RCCs) that may be encountered to modify treatment strategies, instrument selection, and materials to align with the specific case at hand. A substantial body of research has been dedicated to the examination of internal tooth anatomy at the level of individual teeth or groups of teeth, and this includes the maxillary central incisor (MxCI) [1,5,6,7,8,9,10,11,12,13,14,15,16,17,18,19]. Traditionally, techniques such as staining and clearing, light microscopy, and conventional radiography have been used to visualize internal morphology. However, these techniques are severely limited in terms of three-dimensional representation [1,4,6,7,9]. Modern imaging techniques, such as cone beam computed tomography and micro-computed tomography, have transformed the examination of the internal root canal anatomy [11,12,13,14]. While CBCT can be used *in vivo*, µCT remains the gold standard for detailed *ex vivo* analyses of human teeth. The imaging technique provides accurate, non-invasive, and reproducible visualization of the internal tooth structure, which is of value when examining complex anatomical variations [14].

µCT is an efficient tool for examining dental structures and is widely used in dental research to analyze the morphology of pulp chambers and root canals [20]. Reconstructed images also enable the study of morphological features of the pulp chamber, volume ratio in areas such as the pulp horn or pulp chamber floor, and analysis of canal opening diameters [21]. Regarding root canal morphology, the volume and surface area of individual root canals, their configuration and diameter at various measurement points, and the curvature of a root canal can be modeled [20,21,22,23]. In addition, µCT allows three-dimensional reconstruction and visualization of tooth components [24].

µCT enables three-dimensional reconstruction of the various tooth components. In most cases, the tooth structure is depicted transparently, while the pulp chamber and root canal system remain opaque and the pulp is highlighted in color [13,14]. Both the external and internal morphology of the tooth can be precisely reconstructed and the relationship between the complex macro-morphology of the crown and root analyzed [25]. These image data serve as a basis for the investigation of root canal anatomy, including preclinical training in endodontology and the mathematical modeling of tooth morphologies for research purposes [20,21,22,23,24]. µCT is considered a non-invasive, high-resolution, and reproducible method and is regarded as the gold standard for *ex vivo* studies in research into internal tooth morphology [20,24,26].

With the help of modern 3D software, it provides detailed insights into root canal morphology that often cannot be adequately described using classic classification systems such as those from Vertucci [1] and Weine et al. [27]. The system developed by Briseño Marroquín et al. [28] is therefore used as a supplement, dividing the root into thirds (apical, middle third, coronal) and indicating the number of main foramina using a four-digit coding system. These classifications consider different numbers of specific anatomical features, such as accessory foramina, root canals, or anastomoses. A comprehensive understanding of root canal anatomy is essential for the success of both nonsurgical and surgical endodontic treatments. It helps clinicians choose the appropriate instruments and techniques and optimally adapt the treatment to the individual anatomy of the tooth. These advances in imaging are of critical importance given that up to 20% of maxillary anterior teeth may exhibit anatomical modifications, such as the occurrence of accessory canals that are challenging to detect using conventional methods [7,8,13,14]. Such variations increase the risk of failure in endodontic treatment and underscore the necessity for accurate preoperative imaging to identify potential challenges.

The objective of the current study was to investigate the internal morphology of maxillary central incisors and possible different RCCs in a Swiss–German population using µCT, which, to the best of the authors’ knowledge, has not yet been investigated.

The null hypothesis stated that the root canal configurations of maxillary central incisors in a mixed Swiss–German population show no significant variation and do not display distinct, identifiable patterns. The insights of the present study should facilitate the development of more precise diagnostic and therapeutic approaches that better account for individual anatomical conditions, thereby enhancing the long-term success rate of endodontic treatments.

## 2. Materials and Methods

### 2.1. Tooth Sample

Human maxillary central incisors (MxCIs) from Switzerland and Germany were utilized in this study. The 112 teeth from private dental practices in both countries for unknown reasons were extracted unrelated to the present study. Two observers (K.S.F.O., T.G.W.) examined the teeth independently to identify unique anatomical features and determine whether they could be unequivocally classified as maxillary central incisors [29]. In the case of an inconclusive determination, advice was sought from a third independent observer (A.L.W.). The same observers and the same procedure were also used to examine the root canal configurations and anatomical features. All teeth that were investigated in this study were declared as “excess material”; thus, they could be used for scientific purposes without requiring any additional approval of the corresponding ethics committee (Contract General Terms [AVB], §14 Organ explantation/further use of body material, Status: 1 April 2017). The required sample size was calculated for teeth extracted between 1 June and 31 December 2022, using a proportion test with a 95% confidence level and an expected prevalence of 90%. To mitigate the potential for bias due to unusable samples or artifacts, the original sample size of *n* = 97 was increased by 15% [30]. Teeth exhibiting incomplete root development were excluded from the study, as were teeth displaying resorption, fractures, extensive fillings, radicular or coronal caries, or previous endodontic treatment. The remaining teeth were cleaned of soft tissue debris and calculus using an ultrasound device (Pieton 150; EMS Dental, Nyon, Switzerland) and then preserved in a solution of 2% chloramine (Sigma-Aldrich, St. Louis, MI, USA). For µCT examination, teeth were embedded in tubes with cotton rolls and transferred back into the chloramine solution, and the tubes were hermetically sealed with paraffin foil.

### 2.2. Morphological Analysis Using µCT

The maxillary central incisors were scanned in a desktop µCT unit (μCT; SCANCO Medical AG, Brüttisellen, Switzerland) at 70 kV and 114 mA, generating a total of 800 to 1200 cross-sectional images per tooth. To visualize the root canal anatomy, the cross-sectional images were reconstructed in three dimensions using VGSTUDIO Max 2.2 software (Volume Graphics GmbH, Heidelberg, Germany) and displayed in different colors. The pulp chamber and root canal system were colored red, the enamel was colored white, and the dentin was displayed transparently, thus enabling optimal assessment. The root canal anatomy was evaluated in accordance with the classification system proposed by Briseño-Marroquín et al. [28]. This classification employs a four-digit code that subdivides the root canal structure into three sections: coronal, medial, and apical. The fourth digit indicates the number of main foramina with a diameter of at least 0.2 mm. Accessory root canals were defined as additional canals that do not originate from the same main canal at the apical end and whose diameter is less than 0.2 mm [28]. The root canal configuration, accessory canals, and accessory foramina were evaluated in both relative and absolute numbers.

## 3. Results

The root canal configurations (RCCs) are presented in Table 1.

The maxillary central incisors (MxCIs) in the current study were single-rooted. The most frequent RCC according to the classification system of Briseño Marroquín et al. [28] was 1-1-1/1 (Vertucci I, 97.3%). Otherwise, only one other RCC of 1-1-1/2 (2.7%) was observed, which could not be described by the classification methods of Vertucci [1] or Weine et al. [27]. Overall, 40.2% of the 112 MxCIs examined had accessory canals in the middle (27.7%) and apical thirds (12.5%) of the specimens (Table 2).

The number (*n*) and mean (%) of accessory foramina observed in the coronal, middle, and apical thirds of the maxillary central incisors are shown in Table 3.

**Figure 1:**
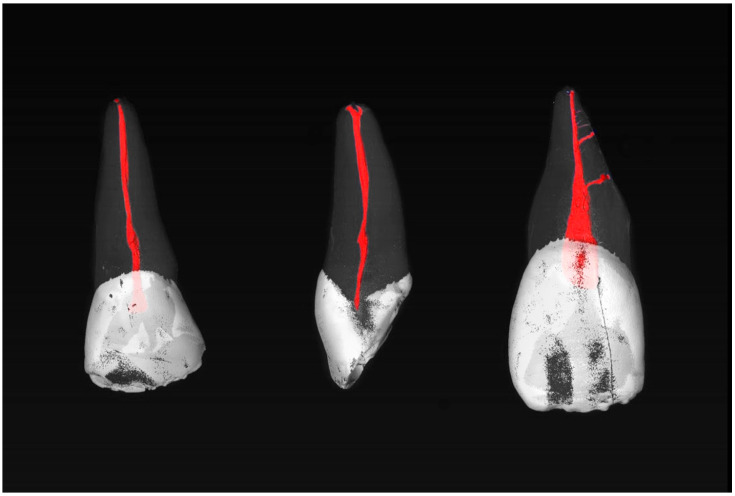
µCT images of maxillary central incisors with a 1-1-1/1 RCC (**left**) and 1-1-1/2 RCC (**center**) without accessory canals, and a 1-1-1/1 RCC (**right**) with two accessory canals in the middle root third.

**Figure 2:**
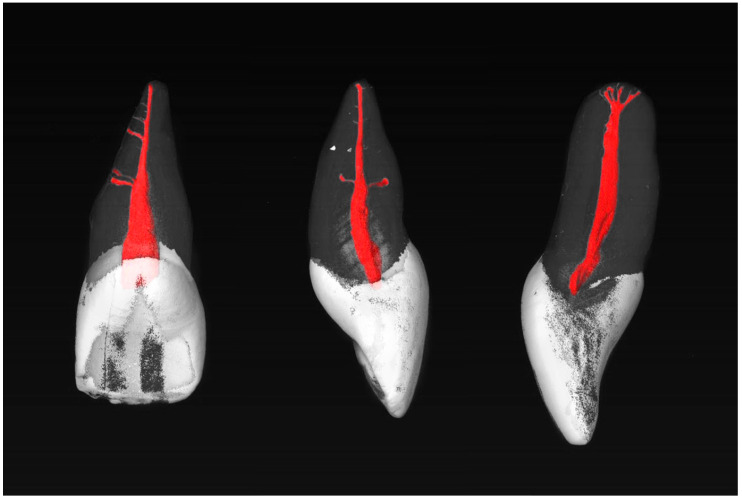
µCT images of maxillary central incisors with an RCC of 1-1-1/1 with four accessory foramina, including two accessory canals in the middle and apical thirds of the root (**left**), two accessory canals in the middle third of the root (**center**), and three accessory foramina in the apical root third (**right**).

Most commonly, the MxCIs had no accessory apical foramina (85.7%). One (8.0%), two (3.6%), three (0.9%), or four (1.8%) could be observed (diameter > 0.1 mm). Examples of the MxCI specimens are shown in Figure 1 and Figure 2.

## 4. Discussion

The objective of the present study was to investigate the internal morphology of the maxillary central incisors (MxCIs) of a mixed Swiss–German population. A detailed description of the various RCCs and the morphology of the apical region of these teeth should be provided to clinicians. The use of µCT enabled the entire root canal system to be visualized in detail. The most prevalent RCC was identified as 1-1-1/1 (Vertucci I), observed in 97.3% of cases, followed by 1-1-1/2 (2.7%). The presence of accessory canals was identified in 40.2% of the specimens, most of these located in the middle root third (22.3%). The results demonstrate that most maxillary central incisors exhibit a simple RCC, yet a considerable proportion of samples displayed the presence of accessory canals. This finding underscores the necessity for a comprehensive approach during root canal treatment, particularly in situations where therapeutic intervention is required. In such cases, the importance of ensuring effective irrigation and obturation of the root canal system cannot be overstated, as this helps to prevent the recurrence of periapical infections. The results thus showed a significant variability in root canal configurations, confirming the hypothesis that the Swiss and German populations have characteristic morphological patterns. These findings contradict the null hypothesis, which had postulated the absence of recognizable differences.

The results of this study are broadly consistent with previous research on the root canal morphology of the MxCI, with 100% of the investigated teeth exhibiting a Vertucci (Ve) Type I configuration (Br 1-1-1/1). Sert and Bayirli reported a Ve Type I configuration (Br 1-1-1/1) in 98–99% of cases in a Turkish population, with slight variations depending on gender [9]. A Ve Type II RCC (Br 2-1-1/1) was identified in 1% of female samples and a Ve Type IV (Br 2-2-2/2) RCC in 2% of male samples. Similarly, Weng et al. [10] identified a Ve Type I (1-1-1/1) RCC in 95.8% of teeth in a Chinese subpopulation, with an increase to 4.2% for a Type II (Br 2-1-1/1) RCC. The results confirm the general assumption that the MxCI is the tooth with the least anatomical variability, with the Ve Type I RCC (Br 1-1-1/1) being the most prevalent [1,7,9,10]. However, there are also reports of rare variations, such as teeth with two roots or several root canals [31,32,33,34]. Regarding accessory canals, our findings align with other studies that have similarly reported an accumulation of accessory canals in the apical region [1,7].

One of the strengths of the present study is the utilization of µCT, allowing for three-dimensional visualization of the root canal system with high resolution. In comparison to conventional imaging techniques, such as conventional radiography, this method also offers a more detailed visualization than other three-dimensional imaging methods, including CBCT [35,36]. This is particularly evident in the visualization of accessory canals and apical foramina [35,36]. To the best of the authors’ knowledge, this is the first study to examine the root canal configuration of maxillary central incisors in a mixed Swiss–German population using µCT. Another strength is the sample size, which provides a robust data basis. One limitation of this study is that it was conducted in a laboratory setting, potentially limiting the generalizability of the findings to clinical conditions and their relevance in clinical settings. Moreover, the lack of information regarding age, sex, and the precise condition of the teeth limits the interpretation of the results. Regarding prevalence, distortions may have occurred due to the potential for teeth with additional foramina or accessory canals to have been extracted more frequently and therefore included in the sample. Additionally, the number of accessory canals and accessory foramina may have been influenced by artifacts despite proper storage. Another potential limitation of this study is the exclusive use of the three specific classification systems of Vertucci [1], Weine et al. [27], and Briseño-Marroquín et al. [28] that, although established and widely used [37], may not fully capture the complex variability of root canal anatomy. Alternative systems such as Ahmed et al.’s [38] could provide valuable additions by offering additional or more differentiated insights into anatomical features [39], even if the method used by Briseño-Marroquín et al. [28] has been established as useful [37].

The results of the current study support previous research on the root canal morphology of the MxCI, particularly regarding the prevalence of the Ve Type I RCC (1-1-1/1). However, it is recommended that the findings be interpreted with caution, given the absence of data on demographic variables such as age and sex. This may restrict the applicability of the findings to other populations. Moreover, the examination of the teeth was conducted in an *ex vivo* setting. This approach may have resulted in the omission of certain clinical challenges that could potentially arise *in vivo*. Notwithstanding these limitations, this study offers valuable insights that can be integrated into clinical considerations for root canal treatment of MxCIs, particularly regarding the prevalence of accessory canals and apical foramina. These results emphasize the clinical importance of recognizing anatomical variations, as they have a direct impact on treatment strategies and highlight the need for individualized approaches to improve long-term outcomes.

## 5. Conclusions

Considering the limitations of this *ex vivo* study on the internal morphology of maxillary central incisors (MxCIs) in a Swiss–German population, the following conclusions can be drawn:

- Two different RCCs were observed with 1-1-1/1 (Ve I, 97.3%) and 1-1-1/2 (2.7%).
- Most MxCIs had only one main foramen, while accessory foramina were found in 14.3% of cases.
- Accessory canals were found in 40.2% of the samples, with these being localized predominantly in the middle (22.3%) and apical thirds (12.5%) of the root.

## Figures and Tables

**Table 1:** RCCs of 112 maxillary central incisors using µCT. The different RCCs are listed according to the Briseño-Marroquín et al. [28], Weine et al. [27], and Vertucci [1] classifications, whereby the latter two have no variant for 1-1-1/2. The classification of Briseño-Marroquín et al. describes the root from the coronal to middle to apical part. The last number after the backslash describes the number of main apical foramina [28].

Root Canal Configuration			Teeth (*n*)	Teeth (%)
Briseño-Marroquín et al. [28]	Weine et al. [27]	Vertucci [1]		
1-1-1/1	I	I	109	97.3
1-1-1/2			3	2.7
**Total**			**112**	**100.0**

**Table 2:** Number (*n*) and mean (%) frequency of accessory root canals observed in the coronal, middle, and apical thirds of maxillary central incisors (*n*^total^ = 112).

Accessory Canals	(*n*)	(%)
**None**	67	59.8
**Coronal**	0	0.0
**Middle**	31	27.7
**Apical**	14	12.5

**Table 3:** Number (*n*) and mean (%) frequency of accessory foramina observed with a diameter smaller than 0.2 mm in the apical third of the root (*n*^total^ = 112).

Accessory Foramina	(*n*)	(%)
**None**	96	85.7
**One**	9	8.0
**Two**	4	3.6
**Three**	1	0.9
**Four**	2	1.8
**Total**	**112**	**100.0**

## Data Availability

Data are contained within the article.
